# Transgender children and young people: how the evidence can point the way forward

**DOI:** 10.1192/bjb.2022.3

**Published:** 2023-04

**Authors:** Philip Graham

**Affiliations:** University College, London, UK

**Keywords:** Transgender, young people, children, research, treatment

## Abstract

The development of gender identity in children from around the age of 3 years is described. Wishes for transgender identity are distinguished from gender-atypical behaviour. Reasons for the recent rise in transgender referrals in the early teen years are discussed. The now widely used protocol developed by the Amsterdam group for assessing transgender children and young people and, where appropriate, offering them puberty blockers, cross-sex hormones and sex reassignment surgery is described. Evidence for the effectiveness of this approach is considered. The competence of young people to give consent to these procedures is discussed. Finally, proposals are made for topics urgently requiring further research.

Children first begin to develop a sense of biological gender at around the age of 2 to 3 years.^1^ At this age, they are able to label pictures of boys and girls according to typical presentations of heteronormativity. At 4 years, boys understand that it is the possession of a penis that marks them out as biologically male and girls understand it is the lack of a penis that means they are biologically female. By this age, children have a sense of the stability of biological gender, an understanding that it remains constant with time. From this point up to the age of 6 or 7 years, their judgement of gender in pictures of clothed children is heavily influenced by appearance so that they label boys pictured in dresses as girls and boys with long hair as girls. By 7 years they recognise biological sex as constant and independent of external appearance.^1^

By the age of 7 years, therefore, children understand three different concepts related to sex/gender identity: biological sex, self-perceived gender identity and social gender identity. They understand that they and others are biologically male or female, that they and others have a sense of their own gender identity as male or female and that they and others, depending on their appearance and clothing, are usually perceived by others as male or female. As they develop into adolescence and adulthood, people recognise that, with the use of hormones and surgical interventions, some features of biological sex can be changed. Both self-perceived gender identity and social gender identity may also undergo change.

The great majority of young children develop a self-perceived gender identity consonant with their gender assigned at birth, but some, from the age of 3 or 4 years, develop a self-perceived gender identity which is other than that assigned at birth. This sense of another gender identity can be accompanied by a feeling of discomfort or gender dysphoria. There are many autobiographical examples of the first awareness of gender dysphoria. The best known is that written by Jan Morris, who lived as a highly successful male journalist under the name of James Morris until her mid-30s when, following treatment with hormones, she underwent a surgical reconstruction and thereafter lived as a woman.^2^ Jan Morris describes very clearly the onset of her gender dysphoria:^2^ ‘I was three or perhaps four years old when I realized I had been born into the wrong body and should really be a girl. I remember the moment well, and it is the earliest memory of my life’ (p. 1). Her sense of discomfort with her assigned gender at birth persisted throughout her childhood, adolescence and early adult life. She describes how, when in role as a young man, she used to pray ‘please God make me a girl’ (p. 39). Gender dysphoria persisted throughout her marriage and parenthood. It was only in her late 30s, after she had had gender reassignment surgery, that she felt at ease.

The majority of prepubertal girls and boys have a clear sense of their own gender identity as female or male. This is nearly always consistent with their gender assigned at birth; in some, like Jan Morris, it is not. In a study of adolescents who had been referred to a gender identity clinic in earlier childhood, Steensma et al were able to show that a high proportion of prepubertal children with gender dysphoria did not continue to show such dysphoria after puberty,^3^ a finding that had previously been reported by the same group.^4^ Further, children who had shown gender-atypical behaviour (see below) without intense gender dysphoria did not generally show gender dysphoria in adolescence. Those with gender dysphoria who had been assigned a female gender at birth were less likely to desist than those assigned a male gender. Those who persisted were much more likely to have a homosexual or bisexual orientation.

A sense of gender identity must be distinguished from the presence of gender-atypical behaviour, which may occur with or without gender dysphoria. Gender-atypical behaviour (boys behaving like girls and having interests generally regarded as feminine and *vice versa*) is not uncommon in the general population. In a total population study, using a standardised instrument, Golombok et al were able to identify 112 boys and 113 girls aged 3.5 years who showed gender-atypical behaviour to an extreme degree.^5^ This represented about 2.2% of the population studied (S. Golombok, personal communication, 5 Jan 2021). Especially for girls, there was considerable continuity between gender-atypical behaviours at 3.5 years and such behaviour at the age of 13 years. These investigators do not report whether any of the children in their study were referred for gender dysphoria. The prevalence of 2.2% for gender-atypical behaviour needs to be contrasted with the much less frequent prevalence of 1 per 6800 Dutch adolescents aged 12 to 18 years who requested medical help for gender dysphoria.^6^

## Gender dysphoria and the onset of sexual feelings

Between 9 and 13 years of age, children start to experience sexual feelings arising from their genitalia. This onset of sexual feelings coincides with biological changes known as gonadarche. At this point, as a result of changes in the hypothalamus and pituitary, the gonads begin to secrete the sex hormones, testosterone and oestradiol, in relatively small quantities. This results in a modest growth of hair around the pubes and in the armpits and growth of the penis and breasts respectively. Spontaneous penile erections and clitoral excitement occur. Around 2 years later, positive feedback occurs in the hypothalamo–pituitary–gonadal axis which stimulates the testes to produce much larger amounts of testosterone and the ovaries to secrete more oestradiol, leading to menstruation. These hormonal changes also result in much more intense experience of sexual desire.

In the majority of children, sexual attraction is heterosexual but around 10% of 16- to 44-year-old adults report some previous sexual contact with a member of the same sex.^7^ Most of those who experience homosexual attraction are not transgender. Usually, they have not even shown gender-atypical behaviour; they have been typically masculine, if boys, and feminine, if girls. Transgender boys usually, but not always, feel attraction to others of the same natal sex, i.e. they have homosexual feelings, and transgender girls similarly feel attracted by others of the same natal sex. Inevitably, these sexual feelings are often associated with some degree of confusion and uncertainty. For most transgender boys and girls, however, homosexual feelings have the effect of confirming the child in their transgender role: ‘If I'm really a girl, it isn't surprising I'm attracted to boys’, a transgender natal boy might say to himself and *vice versa* for girls. But some transgender children develop sexual attraction for others of the opposite natal sex, again with the creation of confusion and uncertainty over the transgender role.

## Adolescence and gender identity

Adolescence is a social construction, i.e. it is a phase of life defined by society.^8^ In Western society, it is regarded as beginning at the onset of biological puberty. Its end is not, however, defined biologically, but usually by a social criterion such as the age at which the individual develops significant autonomy. In practice, most psychologists, clinicians and members of the general public equate adolescence with the teen years, from 13 to 19, although many young people are well into biological puberty by 13 years and will have completed the biological changes of puberty well before 19 years. Recently, Sawyer and colleagues in an influential article have argued for an expanded and more inclusive definition of adolescence corresponding with the longer period of transition from childhood to adulthood now experienced by young people in Western society. They suggest that the period of 10 to 24 years is more consistent with this experience.^9^ It is of relevance that there is considerable variation in ages at onset and termination of biological puberty, some young people normally starting at 10 or 11 years old and others not completing puberty until their later teen years. Relatively recent neuroscientific studies have pointed to the fact that rapid biological changes occur in the brain during the teen years,^10^ but these are by no means specific to this phase of life.^11^

The general public regard various behaviours as characteristic of adolescence. These may be summarised as impulsiveness, a tendency to take risks, moodiness and fractious relationships with parents. The public image of adolescents accords with this view of ‘the typical adolescent’. It is certainly the case that some teenagers show these characteristics, but population studies suggest that they make up no more than about 10–15% of this age group,^12^ although they are certainly the most conspicuous. Another important and, in the context of this article, the most relevant feature of adolescence is thought to be self-questioning about identity. Young people of this age are seen as preoccupied with the question ‘Who am I?’, a question relating to all aspects of their identities, including their gender and sexuality. Such self-questioning is not experienced in intense form by most teenagers. The prevalence of ‘identity problems’ was found to be 14.3% in a group of 15- to 18-year-old American high school students^13^ and a similar prevalence of ‘identity distress’ was found in a study of Flemish adolescents and young people aged 14–30 years.^14^ The considerable increase in exposure of teenagers in the past 10 to 15 years to social media replete with references to gender identity would make it surprising if there had not been at least some increase of such self-questioning and confusion in this area.

## Teenage presentation of transgender

Clinics serving the adolescent transgender population observed a change in the referral pattern after about 2005. Most notably, the gender identity clinic in Toronto, Canada, reported a dramatic increase in referrals at that time.^15^ At the Portman Clinic in London (part of the Tavistock and Portman NHS Trust) referrals increased very significantly from 2009 to 2016.^16^ At the Tampere University Hospital, Finland, referrals between 2011 and 2013 far exceeded the number expected from the findings of epidemiological studies.^17^ This had not been the case previously. There were two other changes in the referral pattern over this period. First, previously, roughly equal numbers of boys and girls had been referred, whereas the increase was associated with much higher numbers of those who had been assigned female gender at birth. Second, previously, the rates of mental ill health among referred children had been about the same as in the general population,^18^ whereas now much higher rates of psychiatric disorder, including autism, were reported.^14,16^

It is therefore clear that from 2005 in Toronto and a few years later in other centres, the characteristics of patients referred to transgender clinics in their early and mid-teen years changed very significantly. In considering the reasons for this new pattern, Aitken et al^15^ suggest that one possibility is that, during this period, societal factors made it easier for gay and lesbian youth and their families to seek clinical care. It could be argued, those authors say, that it became easier for girls to ‘come out’ than boys. It might therefore be easier for girls to opt for a transgender identity. Although there is no evidence to this effect, transgender natal girls who found themselves attracted to girls at puberty might have also found it easier to come out as transgender than hitherto. This implies that the increased presentation at adolescence was of girls who had experienced gender dysphoria since their early years. There is another possibility. It is that girls in their teens who are showing mental health problems for other reasons might, searching for an answer to their identity problems or distress, be influenced by social media to question for the first time their gender identity and to see gender change as an answer to their mental dilemmas. This might be more likely if they had previously shown ‘tomboyish’ behaviour. This possibility has been suggested in considering reasons for an increase in referrals of natal girls to a gender identity service between 2009 and 2016.^15^ However, both these possibilities remain hypothetical at present and the reasons for the increase in referrals to transgender clinics is unknown.

Although one should not draw conclusions from a single case, it is of interest that one of the claimants in a judicial review brought about because they felt they had been inappropriately treated with puberty blocking drugs gives an account of her transgender development very much in accord with this second possibility. The claimant described a highly traumatic childhood in which she showed many gender-atypical behaviours: ‘*From the age of 14* she began actively to question her gender identity and started to look at YouTube videos and do research on the internet about gender identity disorder and the transition process’ (para. 78).^19^

Although some cases of first presentation of transgender in the early teen years may arise from so-called adolescent identity problems or identity distress, it is likely that others do occur because the young person has been reluctant to come out as transgender beforehand, even though gender dysphoria has been present from the early years. Further, it is well established that such reluctance may persist well into adulthood, so that there are a number of recorded cases of people who have waited until their 30s or 40s to make this decision.^20^

There is a need for both quantitative and qualitative research to investigate the early histories of girls referred with gender dysphoria for the first time in adolescence. Such research should include interviewing parents about their children's early years.

## Treatment

Life for children who are transgender from their early years can be challenging. At home, they have to try to communicate how they feel to potentially sceptical parents. At school, they are likely to experience disbelief, mockery and bullying. To cope they need resilient personalities as well as sensitive and understanding parents who are able to explore and talk openly about their children's feelings with acceptance and without trying to influence decisions one way or another. For, as we have seen, although some prepubertal children persist in their transgender identity, in the course of time many will, for reasons we do not understand, desist.^3^ It is remarkable that most children who have been transgender from a young age reach adolescence without developing a higher-than-expected rate of significant mental health problems.^17^

Many prepubertal children and their parents will benefit from having available a sympathetic counsellor, psychotherapist or other mental health professional. This will allow exploration of the reasons for the presence of gender dysphoria. Material from voluntary organisations such as Mermaids may be helpful, but parents of young children need to monitor this to ensure that their children are not being encouraged to persist, but are just accepted for what they are at the present time. Difficult decisions about changes of name and the use of toilets need to be negotiated with hopefully sympathetic, open-minded teachers.

As puberty approaches, difficult decisions have to be made. The Amsterdam group has been offering transgender adolescents puberty blockers for 30 years, their first case having been treated in 1991.^21^ The group has pioneered an approach to assessment and management of gender dysphoria. It has produced a protocol for medical treatment of transgender children and adolescents that has been widely followed,^22^ for example in Italy, Canada, the USA and the UK. The protocol is summarised below and in Box 1:

- Psychological counselling for children and parents starts well before any medical treatment is considered and continues while such intervention is being administered.
- Once Tanner stage 2–3 is reached, and not before, gonadotropin-releasing hormone analogues (GnRHa) are prescribed where there is a clear indication that this is the appropriate course. This medication is given to block pubertal changes, so that the bodily changes rejected by the young person do not occur. Such treatment is only offered to children and young people aged 12 years and older who have intense gender dysphoria and no significant mental health problems. Informed consent by the young person and by the parents is required. The purpose of the use of puberty blockers is to ensure that young people with gender dysphoria do not live through pubertal bodily changes they find abhorrent. Further, the blocking of pubertal changes means that when, as is nearly always the case, transgender adults choose to have at least some degree of gender reassignment surgery, some procedures, particularly bilateral mastectomy for those assigned female gender at birth, will not be necessary.
- With careful assessment and selection, a very small minority of young people prescribed puberty blockers (between 1.4 and 3.5%) change their minds and do not wish to proceed further.^23^ For the large majority who do wish to proceed, around the age of 16 years or older, cross-sex hormones are prescribed. For this treatment to be started, the young person must be living in the role of the preferred gender. Again, informed consent by the young person and, preferably, the parents is required.
- At the age of 18 years or older, those (again the great majority) who meet eligibility criteria can begin the process of gender reassignment surgery. Such surgery occurs variably according to the degree and at the pace desired by the individual concerned.

Box 1 Management of gender dysphoria^22^

1. Make a full assessment as early as possible
2. Follow with supportive counselling throughout childhood and adolescence
3. Subsequent interventions should only take place with informed consent, first by parents and then by the young person, with reflection before each phase
4. If intense gender dysphoria persists, consider using puberty blockers at Tanner stages 2–3
5. Consider use of cross-sex hormones at age 16
6. At age 18–19 and subsequently, consider gender reassignment surgery

## Effectiveness of treatment

The aims of treatment are twofold:

- to explore with the child or young person with gender dysphoria the reasons for their discomfort with their gender assigned at birth and to consider alternative ways forward, including living in the role of their birth-assigned gender or pursuing medical intervention that will enable them to transition;
- in those who choose to live in their preferred transgender role, to start treatment, pausing for reflection before each step, first with puberty blockers, then with cross-sex hormones and finally with gender reassignment surgery to relieve gender dysphoria.

Among those who opt for medical treatment, the degree of success of intervention is measured by the absence of gender dysphoria and mental health problems and by the presence of psychological well-being. Ideally it would be possible to quote findings from a number of controlled trials of each of the interventions. Given the impracticability of obtaining agreement from children and young people with intense gender dysphoria to participate in controlled trials, the findings from uncontrolled but carefully conducted studies provide the main evidence for effectiveness.

There have now been a number of such uncontrolled studies, in which patients have been followed up to see whether their physical and psychological states have improved or deteriorated after the use of puberty blockers alone^24–26^ and puberty blockers followed by cross-sex hormones followed by surgery.^27–29^ The most recently published study of the effects of puberty blockers was reported from the Portman Clinic, London.^30^ This study reported on the short-term outcome over 2 years of 44 children and young people aged 12 to 15 years when they started treatment with puberty blockers. Overall, the patient experience was positive. Although there were some children who showed some negative outcomes in mood and quality of relationships with family and friends, the majority showed positive change. There was no change in the rate of parent- or child-rated behaviour problems or risk of self-harm. All adverse effects, when they occurred, were mild. In line with other studies, only 1 of the 44 children and young people treated with puberty blockers did not go on to request cross-sex hormone treatment.

All the studies quoted above have provided valuable information. In all cases, there has been benefit from the interventions for the majority and an absence of significant harm. The most recent critical review of the use of puberty blockers has concluded: ‘Although large long-term studies with diverse and multicultural populations have not been done, the evidence to date supports the finding of few serious adverse outcomes and several potential positive outcomes. This literature suggests the need for transgender youth to be cared for in a manner that not only affirms their gender identities but that also minimises the negative physical and psychological outcomes that could be associated with pubertal development’.^31^ In all published cases, the majority has reported benefit from the interventions and an absence of significant harm. Where it has been measured, an improvement in psychological well-being has always been found. It is well established that adults who transition ‘experience fewer psychological problems and interpersonal difficulties as well as a strongly increased life satisfaction’ than before the transition and show no wish to revert to their gender assigned at birth.^32^

It should be added that the use of puberty blockers in early adolescence has been strongly criticised.^33,34^ It has been claimed that there has been undue reliance on an affirmative approach (self-identification) in making a transgender diagnosis, that the complexity of the underlying problems of young people presenting as transgender has been inadequately assessed, that a high proportion of those who are treated with puberty blockers regret that they have received this treatment and that the young people who have been treated have not been capable of giving informed consent to treatment that has such profound implications for their future.

## Adverse effects of medical interventions

The effect of puberty blockers is generally, though not universally, regarded as reversible. Their use has been associated with apparently reversible stunting effects on height velocity and bone maturation.^29,35^ General cautions that have been expressed by clinicians about the possibility of irreversibility, such as those by Professor Butler and Dr de Vries quoted in a judicial review,^19^ are no more than one might expect in relation to a large number of interventions in routine use. Caution about possible harm is always an appropriate clinical stance. It should not be taken to mean that the intervention in question should not be used where it is indicated.

There is one undeniable loss that occurs as a result of the use of puberty blockers. The individual does not go through the experience of the ‘normal’ adolescence he or she would have had without their use. However, most transgender young people do not consider this to be a loss or in any way regrettable.

The use of cross-sex hormones exposes the individual to the risk of a metabolic abnormality in about 15% of cases, but the significance of this finding is not clear and it does not seem a contraindication to their use.^36^ Further research is required on the nature of possible metabolic abnormalities arising from the use of cross-sex hormones.

## Informed consent

The competence of young people to give informed consent to the use of puberty blockers and cross-sex hormones is currently a matter of great relevance to clinical management. In UK law, 16 years is regarded as the youngest age at which it can be assumed, on the basis of chronological age, that a young person can give informed consent to a medical procedure. Below that age, it is widely accepted that, in considering whether a young person is capable of giving informed consent, the so-called Gillick principle should be applied. This principle, expressed by Lord Scarman in a 1985 House of Lords judgment and repeated in the above-mentioned judicial review,^19^ is that ‘as a matter of law the parental right to determine whether or not their minor child below the age of 16 will have medical treatment terminates if and when the child achieves sufficient understanding and intelligence to […] understand fully what is proposed’. There is a controversy as to whether, because of the unusually complicated issues involved, children under the age of 16 could ever have the cognitive competence to give consent to puberty blockers or cross-sex hormones. This matter was considered in great detail in the judicial review whose judgment was published in December 2020.^19^ This court decided that young people under 16 years could not give informed consent to the use of puberty blockers. Further, the court ruled that, even in cases where parents give their informed consent and clinicians are in agreement, an application should be made to the courts for authorisation before a child under 16 years can be administered puberty blockers. However, on appeal, this decision was reversed. The Appeal Court decided that the initial judgment had placed an improper restriction on the Gillick test and that it would not be appropriate for an application to the courts to be required before a child could be administered puberty blockers.^37^

There is a need for systematic psychological investigation into the capacity of children and young people to make decisions in this area. Although there is some evidence on the capacity of young people aged 14–16 years to understand medical procedures, there is no evidence relating to the specific question of their understanding of the use of puberty blockers and cross-sex hormones, for example, in comparison with that of older people. Such evidence should be obtained. In the meantime, it would seem reasonable to rely on the findings of Weithorn & Campbell, whose study provides the most relevant data.^38^ These investigators looked at 24 individuals in each of four age groups: 9, 14, 18 and 21 years. They tested their competence to make informed treatment decisions in a series of medical dilemmas, involving conditions such as epilepsy, diabetes and psychological problems. The children, adolescents and young adults were given the nature of the problem, treatments options, expected benefits, possible risks and consequences of failure, and then assessed on how much they understood. The 14-year-olds did as well as the 21-year-olds. The 9-year-olds did distinctly less well. Although it is many years since this study was carried out, until more relevant evidence is produced, there is no reason why its findings should not be regarded as highly pertinent.

## Conclusions

One can conclude from the evidence that gender dysphoria is a relatively rare but well-defined condition, characterised by a strong desire to be of the gender opposite to that assigned at birth and by an insistence that one is, indeed, of the other gender. Affected transgender individuals are usually aware of its existence by the age of 5 years. Gender dysphoria needs to be distinguished from gender-atypical behaviour, where those assigned male gender at birth showed an interest in activities generally preferred by girls and *vice versa*. Marked gender-atypical behaviour occurs in around 2–3% of the population, most of whom are not transgender. Further, many children who show gender dysphoria before puberty do not continue to do so during and after pubertal changes occur. However, if gender dysphoria does persist into adolescence, its intensity tends to increase at this time.

From about 2005 until the present, there has been a considerable, perhaps tenfold, increase in the number of children and young people referred to gender identity clinics. This change has been observed not just in the UK, but in Canada, the USA and Finland. These more recent referrals have differed from previous cases in three ways. More recent referrals have been older, often not presenting until the early teen years. Whereas previously referrals were relatively evenly balanced between those assigned male and female gender at birth, there is now a considerable preponderance of those assigned female gender at birth. Further, whereas previously children and young people with transgender did not show high rates of behavioural and emotional disturbance, this is not the case for recent referrals.

The assessment and management of gender dysphoria has been pioneered by a Dutch group based in Amsterdam. This group has laid down a number of principles of management, which have been widely adopted by gender identity clinics in other countries. The effectiveness of this sequence of interventions is now reasonably well established, with good evidence that it relieves gender dysphoria and usually improves psychological well-being. Physical side-effects may occur but as far as can be ascertained at present, not to a degree where possible harm outweighs benefit. There are, however, unresolved issues concerning the capacity of young people with gender dysphoria to give informed consent to the use of puberty blockers.

There are a number of gaps in knowledge requiring urgent attention. First, it is unclear whether the considerable increase in referrals to gender identity clinics in the past 15 years is due to greater willingness of early affected individuals to come out at this age or whether clinics are dealing with a different population with different needs. There is clearly a need for both quantitative and qualitative research to investigate the early histories of those assigned female gender at birth referred with gender dysphoria for the first time in adolescence. Such research should include interviewing parents about their children's early years. Second, although it is reasonably well established that the use of puberty blockers is not accompanied by serious adverse effects, further research is required on the nature of possible metabolic abnormalities arising from the use of cross-sex hormones. Finally, there is a need for research into the capacity of children and young people, compared with older people, to understand the implications of the use of puberty blockers and cross-sex hormones.

## Data Availability

Data availability is not applicable to this article as no new data were created or analysed in this study.

## About the author

**Philip Graham** is Emeritus Professor of Child Psychiatry in the Institute of Child Health, University College, London, UK.
